# Allogeneic hematopoietic cell transplantation for autoinflammatory disorders

**DOI:** 10.1007/s12185-025-04021-0

**Published:** 2025-06-11

**Authors:** Hirokazu Kanegane, Satoshi Miyamoto, Takahiro Kamiya, Hidenori Ohnishi, Ryuta Nishikomori, Andrew R. Gennery, Takehiko Mori

**Affiliations:** 1 Department of Child Health and Development, Graduate School of Medical and Dental Sciences, Institute of Science Tokyo, 1-5-45 Yushima, Bunkyo-ku, Tokyo, Japan; 2 Department of Pediatrics and Developmental Biology, Graduate School of Medical and Dental Sciences, Institute of Science Tokyo, Tokyo, Japan; 3 Department of Clinical Research Center, Institute of Science Tokyo, Tokyo, Japan; 4 Department of Pediatrics, Gifu University Graduate School of Medicine, Gifu, Japan; 5 Department of Pediatrics and Child Health, Kurume University School of Medicine, Fukuoka, Japan; 6 Great North Children’s Hospital, Newcastle Upon Tyne, UK; 7 Newcastle University Translational and Clinical Research Institute, Newcastle University, Newcastle Upon Tyne, UK; 8 Department of Hematology, Graduate School of Medical and Dental Sciences, Institute of Science Tokyo, Tokyo, Japan

**Keywords:** Autoinflammatory disorder, Hematopoietic cell transplantation, Innate immune system, Immunosuppressive therapy, Refractory inflammation

## Abstract

The understanding of autoinflammatory disorders, which are caused by the dysregulated activation of the innate immune system, has improved with the discovery of new diseases and the expansion of phenotypes. Inflammation can be controlled using immunosuppressive drugs, biological agents, and molecular-targeted therapies. However, some cases remain refractory to treatment, and certain patients experience side effects associated with the long-term use of corticosteroids. Recently, allogeneic hematopoietic cell transplantation (HCT) was reported to improve symptoms in refractory cases. Based on the previous reports, in this review, we discuss the potential of HCT in the treatment of autoinflammatory disorders.

## Introduction

In 1999, Kastner et al. identified variants of the *TNFRSF1* gene as the cause of periodic fever in patients with what had previously been termed familial Irish fever, now known as tumor necrosis factor (TNF) receptor-associated periodic syndrome [1]. Consequently, the concept of “autoinflammatory disorders” emerged, encompassing familial Mediterranean fever (FMF) and hyper-IgD syndrome (HIDS). Autoinflammatory disorders are noninfectious diseases driven by innate immune system dysregulation, and involving the excessive activation of monocytes, macrophages, neutrophils, and other cells. Recent advances in molecular genetic techniques have led to the identification of several genes involved in autoinflammatory disorders [2].

Patients with autoinflammatory disorders are treated with corticosteroids and/or colchicine, as well as targeted biologic therapy, such as TNF inhibitors and molecularly targeted agents, which can suppress inflammation. However, some patients have incomplete inflammation suppression, while others suffer relapses due to secondary drug ineffectiveness. Additionally, the long-term use of corticosteroids poses a risk of adverse effects. Recently, allogeneic hematopoietic cell transplantation (HCT) has been used to ameliorate the symptoms in refractory cases. However, in autoinflammatory disorders, causative genes are often expressed in non-hematopoietic and hematopoietic cells, making it unclear whether HCT effectively regulates inflammation in non-hematopoietic cells. The potential of HCT for treating autoinflammatory disorders is discussed based on the previous reports. HCT has been performed in the autoinflammatory diseases listed in Table 1 [3]. This review describes the indications and outcomes of HCT for autoinflammatory diseases.

**Table 1:** Autoinflammatory disorders for which hematopoietic cell transplantations were performed

Type 1 interferonopathies
Deficiency of adenosine deaminase 2 (DADA2)
OAS1 gain-of-function variant
STAT2 gain-of-function
Proteasome-related autoinflammatory syndrome (PRAAS)
PSMB9 deficiency
Defects affecting the inflammasome
Neonatal-onset cytopenia, autoinflammation, rash, and hemophagocytic lymphohistiocytosis (NOCARH)
Familial Mediterranean fever (FMF)
Proline–serine–threonine phosphatase-interacting protein 1-associated myeloid-related proteinemia inflammatory (PAMI) syndrome
Mevalonate kinase deficiency (MKD)
Cryopyrin-associated periodic syndrome (CAPS)
RIPK1 deficiency
NF-kB-related autoinflammatory disorders (relopathies)
OTULIN-related autoinflammatory syndrome (ORAS)
Haploinsufficiency of A20 (HA20)
Others
Vacuoles, E1 enzyme, X-linked, autoinflammatory, somatic (VEXAS) syndrome
SAMD9L-associated autoinflammatory disease (SAAD)
Sideroblastic anemia with B-cell immunodeficiency, periodic fever, and developmental delay (SIFD) syndrome

## 1. Deficiency of adenosine deaminase 2 (DADA2)

DADA2 is caused by biallelic loss-of-function variants in *ADA2* [4, 5]. Patients present with fever, mild immunodeficiency, polyarteritis nodosa, early-onset stroke, and bone marrow failure, including pure red cell aplasia (PRCA), autoimmune cytopenia, and liver disease. Patients with DADA2 are initially treated with corticosteroids or other immunosuppressants. At present, TNF-α inhibitors are the recommended first-line therapy for DADA2, but they are often ineffective for hematopoietic disorders such as refractory PRCA and immunodeficiencies. To date, 40 patients with DADA2 have undergone HCT [6–8]. The most common indications for HCT were PRCA and neutropenia (11 patients each), while other indications included pancytopenia, autoimmune hemolytic anemia, diffuse large B-cell lymphoma, immune dysregulation, severe aplastic anemia, and severe lymphocytopenia. All but three patients were alive and well at the end of the study, and the overall survival rate was 92.5%. Most patients underwent transplantation after 2015 (n = 32) and were children younger than 18 years of age (n = 29). Half of the HCTs were from HLA-matched unrelated donors. Myeloablative conditioning (MAC) and bone marrow transplant accounted for more than two-thirds of all HCTs.

## 2. Oligoadenylate synthetase 1 (OAS1) gain-of-function variant

The *OAS1* gain-of-function variant is known as OAS1-associated polymorphic autoinflammatory immunodeficiency (OPAID). In 2018, Cho et al. [9] reported heterozygous variants in *OAS1* in three Japanese families with infantile-onset pulmonary alveolar proteinosis and hypogammaglobulinemia. In 2021, Magg et al. [10] reported that OPAID, characterized by recurrent fever, dermatitis, inflammatory bowel disease, alveolar proteinosis, and hypogammaglobulinemia, is caused by gain-of-function variants in *OAS1*. OAS1 is a type I interferon-induced, intracellular dsRNA sensor that generates 2’−5’-oligoadeylate to activate RNase L. The OAS1 variant protein exhibits dsRNA-independent activity, which alters both transcription and translation during interferon-induced expression and is involved in the complex pathogenesis of dysregulation in monocytes, macrophages, and B cells. Two Japanese patients underwent HCT and recovered completely from pulmonary alveolar proteinosis [9, 11]. Alveolar macrophages may be derived from hematopoietic cells. Unfortunately, these patients died of transplantation-related toxicities, such as renal failure and sepsis. The other three patients also underwent HCT [10]. Although one patient died of complications and the other died of chronic graft-versus-host disease (GVHD), the surviving patient achieved clinical correction of the disease phenotype. HCT appears to be curative for patients with OPAID; however, more experience is required to optimize the procedure.

## 3. Signal transducer and activator of transcription 2 (STAT2) gain-of-function

STAT2 is exclusively involved in type I and type III interferon (IFN-I/III) signaling pathways and exhibits unique behavior as both a positive and negative regulator of IFN-I signaling. The opposing functions of STAT2 are exemplified in STAT2 monogenic diseases [12]. Autosomal recessive STAT2 deficiency is associated with an increased susceptibility to severe and recurrent viral diseases [13]. In contrast, homozygous missense substitutions of *STAT2*-R148 residues (R148W/Q) act as gain-of-function (GOF) variants and are associated with severe type I interferon disorders owing to the loss of STAT2 negative regulation [14, 15].

HCT was planned for a 20-month-old patient with the *STAT2*-R148W variant who responded poorly to treatment with dexamethasone and a Janus kinase (JAK) inhibitor. Unfortunately, the patient died of sepsis caused by Gram-negative bacteria during the HCT. Thus, the efficacy of HCT for STAT2-GOF has not been evaluated, and its indications should continue to be discussed.

## 4. Proteasome-related autoinflammatory syndrome (PRAAS)

PRAAS is caused by variants of the gene encoding the proteasome subunit, resulting from impaired proteasome assembly and proteolysis. PRAAS is clinically characterized by early-onset skin disease, hematological and immunological abnormalities, muscle atrophy, joint contractures, and intracranial calcification. The treatment of PRAAS involves lifelong immunosuppression or JAK inhibitors, but the effect is partial, and the side effects are prominent. Because proteasomes are ubiquitously expressed, it is unclear whether HCT is a suitable therapeutic option.

The clinical phenotypes of PRAAS vary between studies. Patients with certain variants of *PSMB10* (c.166G > C, p.Asp56His, and c.601G > A/C, p.Gly201Arg) present with severe combined immunodeficiency, and their clinical features are suggestive of Omenn syndrome [16]. Six de novo patients have been reported to date, all of whom have undergone HCT; however, most have developed severe inflammatory complications, such as GVHD and thrombotic microangiopathy. Two patients died of treatment-related complications, two died of delayed death, and only two survived.

Recently, HCT was reported in a patient with PRAAS type 3 with a compound heterozygous variant of the *PSMB4* gene [17]. The patient presented with periodic fever, cutaneous vasculitis, chronic thrombocytopenia, and abnormal liver function and was indicated for HCT due to treatment-resistant inflammatory condition and treatment-related growth retardation. The first HCT with reduced-intensity conditioning (RIC) was performed at 20 months of age; however, the chimerism of donor-derived lymphoid cells was reduced and relapsed. A second HCT with MAC was performed at 3 years of age: 100% donor-derived chimerism was achieved, and the symptoms improved, except for thrombocytopenia. Growth and development also occurred; however, the patient presented with mild symptoms of lipodystrophy and was suspected of having a relapse of PRAAS symptoms. Patients with treatment-resistant PRAAS can be cured with HCT.

## 5. PSMB9 deficiency

Kanazawa et al. [18] identified a heterozygous missense variant (G465D) in the proteasome subunit beta 9 (*PSMB9*), which encodes β 1i, in two unrelated Japanese infants. In addition to PRAAS-like symptoms, these patients presented with pulmonary hypertension and immunodeficiency, and their clinical phenotype differed from that of typical PRAAS. A mouse model carrying the same variant (*Psmb9*^G156D/+^) exhibited the same proteasomal abnormalities and immunodeficiency phenotypes as the patients. As such, they proposed a new disease concept: PRAAS with immunodeficiency (PRAAS-ID). As patients with PRAAS-ID have an immunodeficient phenotype, HCT may be a viable treatment.

HCT was performed in a patient with a PSMB9 abnormality due to a heterozygous variant (p.G156D) in the *PSMB9* gene. The patient presented with fever, chilblain-like skin rash, myositis, and severe pulmonary hypertension and required the use of an extracorporeal membrane oxygenation (ECMO) device [19]. The symptoms were ameliorated by JAK inhibitor therapy, and cord blood transplantation was performed after RIC at 7 months of age, after weaning from ECMO. Although 63.1% donor-derived chimerism was achieved, JAK inhibitors and vasodilators were discontinued during the 2-year observation period following transplantation, and no relapse of PSMB9-related symptoms, including pulmonary hypertension, was observed.

## 6. Neonatal-onset cytopenia, autoinflammation, rash, and hemophagocytic lymphohistiocytosis (NOCARH) syndrome

Cell division cycle 42 (CDC42) is an intracellular member of the Ras homology GTPase family, which controls cell polarity by regulating the assembly of actin cytoskeleton structures. CDC42 deficiency manifests in a variety of diseases; however, missense variants at the C-terminus result in CDC42-associated autoinflammatory disease, also known as NOCARH syndrome [20]. One study investigated four unrelated patients presenting with neonatal-onset HLH. All patients were treated with glucocorticoids, but three died within the first 2 years of life. One patient was successfully treated with allogeneic HCT from a haploidentical father. Notably, emapalumab (an anti-IFN γ monoclonal antibody) and anakinra (a recombinant human IL-1 receptor antagonist) were administered post-transplantation to prevent HLH relapse. HCT was performed in three of the nine patients with the *CDC42* p.C188Y variant, including the above patient, two of whom survived and were cured [21]. Therefore, early detection of NOCARH syndrome, prompt anti-cytokine therapy, and subsequent indications for HCT are important. Patients with NOCARH syndrome exhibit markedly elevated serum IL-18 levels, which may be associated with their susceptibility to HLH [22]. Emerging anti-IL-18 agents may be useful as bridging therapies for HCT.

## 7. FMF

FMF is a hereditary autoinflammatory disorder characterized by recurrent fever, typically occurring every 4 weeks, along with chest or abdominal pain due to serositis. Clinical diagnosis is straightforward based on symptom presentation and response to colchicine; however, the identification of *MEFV*—the gene encoding pyrin—as the causative gene has enabled a definitive diagnosis [23, 24]. Although FMF is the most common autoinflammatory disorder, only one patient with FMF was treated with HCT and reported improvement in FMF-related symptoms. To the best of our knowledge, this is the first reported case of HCT associated with an autoinflammatory disorder. A 7-year-old girl with congenital dyserythropoietic anemia (CDA) and FMF was identified as a candidate for HCT due to her need for frequent blood transfusions to manage CDA [25]. Following HCT, the patient became transfusion-independent, and her FMF-related symptoms resolved. However, given the availability of safe and effective treatments for FMF, such as colchicine and anti-IL-1 blockade, there remains significant debate regarding the indication of HCT for FMF [26].

## 8. Proline-serine-threonine phosphatase-interacting protein 1-associated myeloid-related proteinemia inflammatory (PAMI) syndrome

PAMI syndrome is a rare autoinflammatory disease characterized by skin vasculitis, hepatosplenomegaly, enlarged lymph nodes, cytopenia, hyperzincemia, and hypercalprotectinemia. This disorder presents a more severe phenotype than pyogenic aseptic arthritis, pyoderma gangrenosum, and acne (PAPA) syndrome, which is caused by variants of the same gene (*PSTPIP1*). The *PSPT1P1* variants p.E250K and p.A230T distinctively contribute to PAMI syndrome [27]. Immunosuppressive and/or anti-cytokine therapies have shown limited efficacy. Monocytes derived from patients with PAPA exhibit IL-18 production in a pyrin-dependent manner when stimulated with IFN-γ [28]. IFN-γ is abundant in the inflamed dermis of patients with PAPA. Clinical improvement was observed in five patients with PAPA following JAK inhibitory therapy.

HCT has been reported as an effective treatment in five patients with PAMI syndrome [29]. No relapse of PAMI syndrome symptoms was observed at a median follow-up of 2.2 years post-HCT. Before transplantation, all patients exhibited cytopenia, and some presented with vasculitis and inflammatory bowel disease. The indications for HCT were disease refractory to medical therapy in four patients and the development of myelodysplastic syndrome in one patient. One patient developed HLH and GVHD post-HCT and subsequently underwent a second HCT procedure. Four patients achieved complete chimerism, while one patient exhibited mixed chimerism with 84% donor-derived cells in whole blood. All patients, including one patient with mixed chimerism, experienced symptom improvement related to PAMI syndrome. Thus, allogeneic HCT may serve as a curative treatment for cytopenia and severe autoinflammation in patients with PAMI syndrome. JAK inhibitors may also be a useful beneficial therapeutic option for these patients, potentially influencing HCT indications.

## 9. Mevalonate kinase deficiency (MKD)

MKD/HIDS is an organic acidemia and rare autoinflammatory disorder. Reports of successful allogeneic HCT outcomes in patients with severe MKD remain limited. Nine patients were enrolled in one study, with HCT indications primarily involving active disease refractory to biologic agents [30]. The treosulfan-based conditioning regimen is widely used, and all but one patient achieved complete remission. Donor sources included cord blood (n = 2), peripheral blood stem cells (n = 4), and bone marrow (n = 4). Two patients required a second HCT due to incomplete or initial engraftment failure. Four patients developed grade II–IV GVHD and two died of transplant-related complications. Allogeneic HCT is an effective treatment for patients with severe MKD; however, treatment-related adverse events and associated mortality remain significant challenges.

## 10. Cryopyrin-associated periodic syndrome (CAPS)

CAPS is an autoinflammatory disorder characterized by skin rash, fever, and inflammation affecting eyes, ears, bones, joints, and meninges. CAPS results from variants in *NLRP3*, which encodes the IL-1 inflammasome protein [31, 32]. Based on its severity, CAPS is classified into familial cold urticaria (mild), Muckle–Wells syndrome (MWS) (moderate), and chronic infantile neurological cutaneous articular (CINCA)/neonatal-onset multisystem inflammatory disease (NOMID) (severe). The quality of life for patients with CAPS has improved with the advent of anti-IL-1β inhibitors [33, 34]. Mori et al. [35] reported a case involving a 30-year-old woman with MWS who developed acute lymphocytic leukemia (ALL), that was refractory to chemotherapy. The patient underwent allogeneic bone marrow transplantation (BMT) from an unrelated donor, which resulted in ALL remission and resolution of CAPS-related symptoms. This suggests that HCT may be beneficial for CAPS. Although the decision to pursue HCT for CAPS should be made with caution, it may be considered in patient with CINCA/NOMID who demonstrate an inadequate response to IL-1β inhibitors. In addition, some patients with CAPS exhibited low-frequency somatic mosaicism [36], complicating post-transplant chimerism analysis. Thus, the role of HCT in cases of somatic mosaicism warrants further investigation.

## 11. Receptor-interacting serine/threonine-protein kinase 1 (RIPK1) deficiency

RIPK1 plays a key role in apoptotic and necroptotic cell death and in inflammatory signaling pathways. Autosomal recessive (AR) RIPK1 deficiency results from biallelic loss-of-function variants in *RIPK1* [37]. This deficiency is characterized by impaired T- and B-cell differentiation, lymphopenia, and reduced production of IL-6, TNF-α, and IL-12. Patients with AR RIPK1 deficiency exhibit inflammatory bowel disease (IBD), polyarthritis, and recurrent infections. Five patients with AR RIPK1 underwent HCT [38]. All had very early onset IBD, with a median age of 3 years (range 1–5 years) at the time of transplantation. All patients received reduced-toxicity conditioning with either treosulfan (n = 4) or busulfan (n = 1). Four patients survived; however, one died due to *Pseudomonas* infection and multi-organ failure. Among the surviving patients, all exhibited improvement in IBD symptoms. Therefore, HCT may be a curative therapy for AR RIPK1 deficiency.

## 12. OTULIN-related autoinflammatory syndrome (ORAS)

ORAS or otulipenia is an autoinflammatory disorder caused by hypomorphic variants in *OTULIN,* which is linked to the NF-κB pathway. Clinical manifestations include skin inflammation and panniculitis, typically presenting in early infancy. A 17-month-old patient with an autoinflammatory disease refractory to prednisone, colchicine, and anakinra (IL-1 inhibitor) underwent HCT from an HLA-matched father [39]. After transplantation, the patient's inflammatory symptoms improved; however, 9 months after HCT, donor chimerism decreased, and clinical symptoms recurred. The recurrent symptoms were controlled using TNF inhibitors. The patient was subsequently diagnosed with ORAS. These findings indicate that ORAS symptoms can be controlled if complete remission is achieved; however, the necessity of HCT remains debated, given the availability of effective TNF inhibitor therapy. Nevertheless, HCT may be considered before treatment resistance develops and irreversible organ damage occurs.

## 13. Haploinsufficiency of A20 (HA20)

HA20 is caused by a heterozygous loss-of-function variant in the *TNFAIP3* gene [40]. Patients present with a Behcet's disease-like autoinflammatory phenotype alongside autoimmune features. Corticosteroids and immunosuppressive agents, including TNF inhibitors, have demonstrated efficacy. Two patients with HA20 underwent HCT due to refractoriness to multiple immunosuppressive agents and cytokine antagonists [41]. The first patient, a 14-year-old boy, underwent HCT from an unrelated donor. After 3 years of follow-up, he exhibited a high degree of chimerism, and his pulmonary and gastrointestinal symptoms improved. The second patient underwent HCT from an HLA-matched non-carrier donor. One year after transplantation, the patient's inflammatory symptoms improved; however, adrenal insufficiency associated with long-term prednisolone therapy persisted.

Because A20 is expressed in both hematopoietic and non-hematopoietic cells, HCT may correct excessive inflammation and autoantibody production by hematopoietic cells. However, HCT may not ameliorate A20 dysfunction in non-hematopoietic cells, such as keratinocytes and intestinal epithelial cells, and future clinical manifestations may result from the dysregulation of non-hematopoietic cells.

## 14. Vacuoles, E1 enzyme, X-linked, autoinflammatory, and somatic (VEXAS) syndrome

VEXAS syndrome is an adult-onset autoinflammatory disease caused by somatic variants in the *UBA1* gene, which are involved in protein ubiquitination, resulting in severe systemic inflammation and blood disorders, such as myelodysplastic syndrome [42]. One therapeutic strategy for VEXAS syndrome is to control hyperinflammation and *UBA1* mutant clones [43]. Various immunosuppressive agents are used to control hyperinflammation; however, JAK and IL-6 inhibitors are more effective than other immunosuppressive agents, whereas ruxolitinib is more effective than other JAK inhibitors. Azacitidine, a hypomethylating agent often used in patients with myelodysplastic syndrome and other malignancies, has also been explored. Allogeneic HCT is potentially the most effective curative therapy for VEXAS syndrome. However, most patients with VEXAS syndrome are older adults and may not be ideal candidates for HCT due to age-related risks and comorbidities.

Thirty-three cases of HCT for VEXAS syndrome have been reported [44]. Most patients (n = 32, 97.0%) were male. The median time from symptoms onset to HCT was 3 years, and the median age was 59 years. The most common *UBA1* variant was p.Met41Thr (11/32, 34.4%). The median variant allele frequency was 56.5%. Peripheral hematopoietic stem cell transplantation (30/31, 96.8%) and human leukocyte antigen-matched unrelated donors (18/32, 56.3%) were the most common. The conditioning regimens varied, with RIC with fludarabine being the most frequently used (12/31, 38.7%). Acute and/or chronic GVHD (18/32, 56.3%) and infection (12/32, 37.5%) were common complications. Overall, 27 patients (81.8%) survived. Of the six patients who died, infection was the cause in four patients. Eleven patients for whom molecular data were available after HCT exhibited complete resolution of the *UBA1* variant. HCT is not only a treatment option: it may also offer a potential cure. However, comorbidities, concomitant hematologic diseases, and the overall risk of GVHD and infection must be fully considered.

## 15. SAMD9L-asssociated autoinflammatory disease (SAAD)

Heterozygous de novo frameshift variants in the *SAMD9L* gene have been identified as a cause of neonatal-onset autoinflammatory diseases and are referred to as SAAD. Patients with SAAD present with neutrophilic panniculitis and progressive B and NK lymphopenia [45]. Four patients with SAAD were reported to have been treated with HCT. The first two patients underwent HCT at 20 and 9 months of age and are currently doing well [45]. The third patient died at the time of transplantation due to pre-transplant lung damage [46]. The fourth patient underwent TCRαβ/CD19-deleted HCT from a haploidentical donor, which improved his inflammatory symptoms but was associated with severe growth retardation and motor development impairment [47]. Neurological symptoms may not improve after HCT, and thus, the indications for HCT in SAAD should be carefully considered.

## 16. Sideroblastic anemia with B-cell immunodeficiency, periodic fever, and developmental delay (SIFD) syndrome

SIFD syndrome is a rare autoinflammatory disorder [48]. Patients with SIFD syndrome harbor biallelic variants in the *TRNT1* gene [49]. Frequent manifestations include microcytic or sideroblastic anemia, recurrent fever, neurological abnormalities, humoral immunodeficiency, gastrointestinal symptoms, and others [50]. Approximately one-third of the reported patients die of disease progression or HCT complications. TNF- α administration and HCT may help alleviate disease symptoms. HCT has been performed in five patients; however, three patients succumbed to complications shortly after HCT. Two patients survived following successful HCT, with complete resolution of symptoms, including fever, immunodeficiency, hematological abnormalities, growth failure, and neurologic deficits. Although HCT is associated with a high risk, it may be a valuable treatment option, as SIFD syndrome is potentially curable.

## Conclusion

As the causative genes of many inherited autoinflammatory diseases are discovered and their pathogenesis elucidated, reports of systemic inflammation improvement after HCT in refractory cases are emerging. Table 2 summarizes the indications and outcomes of HCT in major autoinflammatory diseases. While some primary immunodeficiency diseases such as severe combined immunodeficiency are absolute indications for HCT, not all autoinflammatory diseases are candidates for HCT. However, it is true that HCT can relieve symptoms related to autoinflammation in patients who are refractory to conventional therapy. Of course, the development of immunosuppressive, molecularly targeted, and anti-cytokine agents is underway, and novel agents are expected to be effective for autoinflammatory diseases. Therefore, it is important to identify patients who are candidates for HCT. It is unclear whether HCT can completely suppress systemic inflammation in all autoinflammatory diseases. Furthermore, some patients have died due to transplant-related complications. Therefore, patient selection for HCT should be carefully considered, ideally based on the natural history of the disease under conservative management. Appropriate use of molecular-targeted and anti-cytokine agents as bridging therapy prior to HCT to improve systemic status is also an important strategy. Timing of HCT is also important, as organs damaged by the underlying disease are unlikely to improve with HCT. Further case studies are needed to evaluate the efficacy of HCT in autoinflammatory diseases. In addition, further studies are needed to optimize the indications, timing, and conditioning regimen of HCT.

**Table 2:** Indication and outcome of hematopoietic cell transplantation for autoinflammatory diseases

Disease	Clinical features	HCT indication	HCT outcome	Post-HCT complications
DADA2	Fever, immunodeficiency, polyarthritis nodosa, early-onset stroke, bone marrow failure	Most common: PRCA, neutropenia Others: pancytopenia, AIHA, DLBCL, immune dysregulation, SAA, lymphopenia	37/40 (92.5%) survived	Graft failure
OAS1 GOF variant	Recurrent fever, dermatitis, inflammatory bowel disease, alveolar proteinosis, hypogammaglobulinemia	All cases may be indicated before complications are allowed	1/5 survived	Renal failure, sepsis, chronic GVHD
NOCARH	Neonatal-onset cytopenia with dyshematopoiesis, autoinflammation, rash, HLH	HLH	2/3 survived	
PAMI syndrome	Skin vasculitis, hepatosplenomegaly, lymphadenopathy, cytopenia, hyperzincemia, hypercalprotectinemia	4: refractory to medical therapy 1: MDS	5/5 survived	HLH, GVHD
MKD	Fever, gastrointestinal symptoms, lymphadenopathy, arthralgia, myalgia, skin rash, mucosal ulcers	Failure of anti-inflammatory therapies and very severe disease cases	2/9 survived	Grade II–IV GVHD Unlikely to improve neuromuscular symptoms
HA20	Behcet's disease-like autoinflammatory phenotype, autoimmune diseases	Refractory to multiple immunosuppressants and cytokine antagonist	2/2 survived	
VEXAS syndrome	Severe systemic inflammation, MDS	Younger patients	27/33 (81.8%) survived	Acute and/or chronic GVHD, infection
SAAD	Neutrophilic panniculitis, progressive B and NK lymphopenia	Severe cases	2/4 survived	Growth retardation and motor development impairment
SIFD syndrome	microcytic or sideroblastic anemia, recurrent fever, neurological abnormalities, humoral immunodeficiency, gastrointestinal symptoms	Severe cases	2/5 survived	Transplantation-related mortality

*HCT* hematopoietic cell transplantation, *DADA2* deficiency of adenosine deaminase 2, *PRCA* pure red cell aplasia, *AIHA* autoimmune hemolytic anemia, *DLBCL* diffuse large B-cell lymphoma, SAA severe aplastic anemia, *OAS1* oligoadenylate synthetase, *GOF* gain-of-function, GVHD graft-versus-host disease, *NOCARH* neonatal-onset cytopenia autoinflammation rash and hemophagocytic lymphohistiocytosis, *HLH* hemophagocytic lymphohistiocytosis, *PAMI* proline–serine–threonine phosphatase-interaction protein 1-associated myeloid-related proteinemia inflammatory, *MDS* myelodysplastic syndrome, *MKD* mevalonate kinase deficiency: *HA20* haploinsufficiency of A20, VEXAS vacuoles *E1 enzyme* X-linked autoinflammatory somatic, *SAAD* SAMD9L-associated autoinflammatory disease, *SIFD* sideroblastic anemia with B-cell immunodeficiency periodic fever and developmental delay

## Data Availability

The datasets for this article are not publicly available due to concerns regarding participant/patient anonymity. Requests to access the datasets should be directed to the corresponding author.
